# *catena*-Poly[[[aqua­(5-nitro­benzene-1,2,3-tricarboxyl­ato-κ*O* ^1^)copper(II)]-di-μ-aqua-[diaqua­sodium]-di-μ-aqua] tetra­hydrate]

**DOI:** 10.1107/S1600536810000401

**Published:** 2010-01-09

**Authors:** Yong-Jie Ding, Chun-Xiang Zhao

**Affiliations:** a Department of Chemistry, East China Normal University, Shanghai 200062, People’s Republic of China; b Department of Chemistry, Zhoukou Normal University, Zhoukou 466001, People’s Republic of China

## Abstract

In the heteronuclear coordination polymer, {[CuNa(C_9_H_2_NO_8_)(H_2_O)_7_]·4H_2_O}_*n*_, the Cu^II^ atom is coordinated by six O atoms from five water mol­ecules and one 5-nitro­benzene-1,2,3-tricarboxyl­ate ligand in a slightly distorted octa­hedral geometry. The Na^+^ cation is surrounded by six water mol­ecules in an irregular trigonal-prismatic geometry. The Cu and Na atoms are connected by water bridges, forming an infinite chain. O—H⋯O hydrogen bonds involving the coordinated and uncoordinated water mol­ecules connect the chains into a three-dimensional network.

## Related literature

For general background to the possible applications of metal coordination polymers as microporous hosts for absorption or as catalytic materials, see: Cheng *et al.* (2004 ▶); Yaghi & Li (1995 ▶).
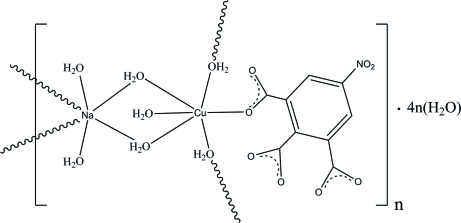


## Experimental

### 

#### Crystal data

[CuNa(C_9_H_2_NO_8_)(H_2_O)_7_]·4H_2_O
*M* *_r_* = 536.82
Triclinic, 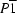

*a* = 6.6480 (13) Å
*b* = 13.124 (3) Å
*c* = 13.531 (3) Å
α = 63.46 (3)°
β = 79.17 (4)°
γ = 82.13 (3)°
*V* = 1035.5 (4) Å^3^
*Z* = 2
Mo *K*α radiation
μ = 1.17 mm^−1^
*T* = 295 K
0.27 × 0.26 × 0.21 mm

#### Data collection

Bruker APEXII area-detector diffractometer
Absorption correction: multi-scan (*SADABS*; Sheldrick, 2005 ▶) *T*_min_ = 0.743, *T*_max_ = 0.791
5466 measured reflections
3696 independent reflections
3113 reflections with *I* > 2σ(*I*)
*R*_int_ = 0.021

#### Refinement

*R*[*F* ^2^ > 2σ(*F* ^2^)] = 0.047
*wR*(*F* ^2^) = 0.133
*S* = 1.02
3696 reflections
280 parameters
H-atom parameters constrained
Δρ_max_ = 0.76 e Å^−3^
Δρ_min_ = −0.76 e Å^−3^

### 

Data collection: *APEX2* (Bruker, 2005 ▶); cell refinement: *SAINT* (Bruker, 2005 ▶); data reduction: *SAINT*; program(s) used to solve structure: *SHELXS97* (Sheldrick, 2008 ▶); program(s) used to refine structure: *SHELXL97* (Sheldrick, 2008 ▶); molecular graphics: *Mercury* (Macrae *et al.*, 2006 ▶); software used to prepare material for publication: *SHELXTL* (Sheldrick, 2008 ▶).

## Supplementary Material

Crystal structure: contains datablocks I, global. DOI: 10.1107/S1600536810000401/ng2712sup1.cif

Structure factors: contains datablocks I. DOI: 10.1107/S1600536810000401/ng2712Isup2.hkl

Additional supplementary materials:  crystallographic information; 3D view; checkCIF report

## Figures and Tables

**Table 1:** Hydrogen-bond geometry (Å, °)

*D*—H⋯*A*	*D*—H	H⋯*A*	*D*⋯*A*	*D*—H⋯*A*
O1*W*—H2*W*⋯O4^i^	0.84	2.06	2.883 (4)	166
O1*W*—H1*W*⋯O5^ii^	0.84	2.19	2.932 (4)	148
O2*W*—H4*W*⋯O3	0.84	1.94	2.706 (4)	151
O2*W*—H3*W*⋯O6*W*^iii^	0.84	1.91	2.741 (4)	169
O3*W*—H6*W*⋯O4*W*^i^	0.84	2.09	2.863 (4)	153
O3*W*—H5*W*⋯O4	0.84	2.01	2.825 (4)	164
O4*W*—H8*W*⋯O3*W*^iv^	0.84	2.11	2.868 (4)	149
O4*W*—H7*W*⋯O3^v^	0.84	2.12	2.902 (4)	155
O5*W*—H10*W*⋯O1^iii^	0.84	2.60	3.174 (4)	127
O5*W*—H10*W*⋯O5	0.84	2.05	2.778 (4)	145
O5*W*—H9*W*⋯O6^vi^	0.84	1.89	2.711 (3)	166
O6*W*—H12*W*⋯O5*W*	0.84	2.00	2.810 (4)	161
O6*W*—H11*W*⋯O7^vi^	0.84	1.91	2.716 (4)	160
O7*W*—H14*W*⋯O2*W*^v^	0.84	1.98	2.788 (4)	160
O7*W*—H13*W*⋯O7	0.84	1.87	2.657 (4)	156
O8*W*—H16*W*⋯O2*W*^v^	0.84	1.85	2.679 (4)	171
O8*W*—H15*W*⋯O6*W*	0.84	1.95	2.774 (4)	167
O9*W*—H18*W*⋯O5^i^	0.84	1.82	2.647 (4)	168
O9*W*—H17*W*⋯O6	0.84	1.99	2.823 (3)	175
O10*W*—H19*W*⋯O5*W*	0.84	1.85	2.674 (4)	166
O10*W*—H20*W*⋯O6^i^	0.84	1.88	2.704 (3)	167
O11*W*—H22*W*⋯O3*W*^v^	0.84	1.85	2.670 (4)	165
O11*W*—H21*W*⋯O4^i^	0.84	1.98	2.776 (4)	158

Symmetry codes: (i) 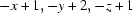; (ii) 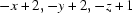; (iii) 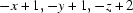; (iv) 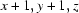; (v) 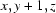; (vi) 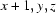.

**Fig. 1.**
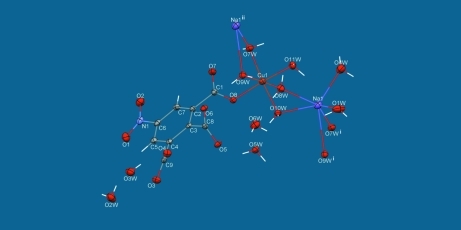
The molecular structure of (NBA) (thermal ellipsoids areshown at 30% probability levels). [Symmetry codes: (i) 1 + x, y, z; (ii) -1 + x, y, z]

**Fig. 2.**
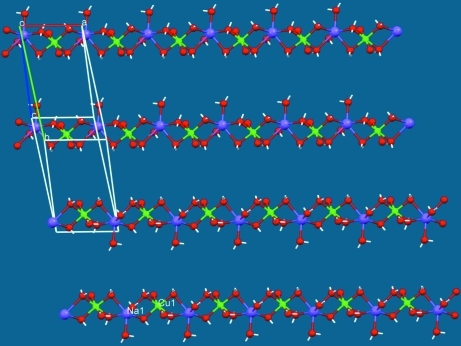
The molecular packing diagram along the a axis (the NBA and water molecules have been omitted for clarity)

**Fig. 3.**
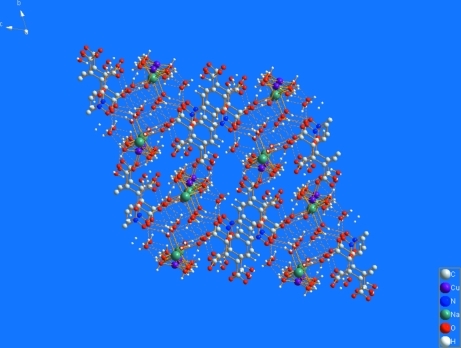
Three-dimensional supermolecular structure is built up through hydrogen bond

## supplementary crystallographic information

### Comment

Recently, there has been much interest in the synthesis of metal coordination polymers, due to their possible application as microporous hosts for absorption or even as catalytic materials (Yaghi *et al.*, 1995; Cheng *et al.*,2004). Herein, we report a new heteronuclear metal coordination polymer with the tricarboxylates, 5-Nitrobenzene-1,2,3-tricarboxylicacid (NBA) as the ligand, the copper (II) and sodium (I) as the metal ions.

As can be seen from the crystal structure in Fig.1, Cu and Na are connected *via µ*-*O*, *O*' coordination of water molecules, which structure is repeating unit along *a *axis, forming one-dimensional infinite chains, which chains along the *a *axis is built up through coordination between NBA, a part of water molecules and Cu(II), Na(I) (Fig.2). Through the forming of hydrogen bonds between chains and water molecules of the interchain, three-dimensional supermolecular structure is formed. The different chains are linked by an extensive hydrogen-bonding network (Table 1, Fig.3), through oxygen atoms of carboxylate and water molecule. Each of the water molecules has at least one hydrogen-bonding interaction, this leads to the formation of a stable three dimensional supramolecular structure.

### Experimental

5-Nitrobenzene-1,2,3-tricarboxylic acid (0.051 g, 0.2 mmol) was added to a solution of copper chloride (0.027 g, 0.2 mmol) (20 mL), the resulting mixture was treated with a solution of NaOH until the pH value come rise to be about 8.The mixture was then stirred continuously for 6 h, and the filtrate was kept in conical flask for about 30 days and blue block crystals were obtained from the solution, dried in vacuum. Yield: 67.6%. Crystal of the title compound suitable for single-crystal X-ray diffraction was selected directly from the sample as prepared.

### Refinement

All C-bound H atoms were placed in calculated positions, with C—H = 0.93Å for phenyl H, and refined as riding, with *U*_iso_(H) =1.2*U*_eq_ (C) for phenyl H. The water H-atoms were placed in chemically sensible positions on the basis of hydrogen bonding but were not refined, with *U*_iso_(H) = 1.5*U*_eq_(O).

### Crystal data

**Table:**

[CuNa(C_9_H_2_NO_8_)(H_2_O)_7_]·4H_2_O	*Z* = 2
*M**_r_* = 536.82	*F*(000) = 554
Triclinic, *P*1	*D*_x_ = 1.722 Mg m^−^^3^
Hall symbol: -P 1	Mo *K*α radiation, λ = 0.71073 Å
*a* = 6.6480 (13) Å	Cell parameters from 2416 reflections
*b* = 13.124 (3) Å	θ = 2.9–27.7°
*c* = 13.531 (3) Å	µ = 1.17 mm^−^^1^
α = 63.46 (3)°	*T* = 295 K
β = 79.17 (4)°	Block, blue
γ = 82.13 (3)°	0.27 × 0.26 × 0.21 mm
*V* = 1035.5 (4) Å^3^	

### Data collection

**Table:**

Bruker APEXII area-detector diffractometer	3719 independent reflections
Radiation source: fine-focus sealed tube	3113 reflections with *I* > 2σ(*I*)
graphite	*R*_int_ = 0.021
φ and ω scan	θ_max_ = 25.2°, θ_min_ = 1.7°
Absorption correction: multi-scan (*SADABS*; Sheldrick, 2005)	*h* = −7→7
*T*_min_ = 0.743, *T*_max_ = 0.791	*k* = −15→15
5466 measured reflections	*l* = −12→16

### Refinement

**Table:**

Refinement on *F*^2^	Primary atom site location: structure-invariant direct methods
Least-squares matrix: full	Secondary atom site location: difference Fourier map
*R*[*F*^2^ > 2σ(*F*^2^)] = 0.047	Hydrogen site location: inferred from neighbouring sites
*wR*(*F*^2^) = 0.133	H-atom parameters constrained
*S* = 1.02	*w* = 1/[σ^2^(*F*_o_^2^) + (0.0826*P*)^2^ + 0.906*P*] where *P* = (*F*_o_^2^ + 2*F*_c_^2^)/3
3696 reflections	(Δ/σ)_max_ = 0.001
280 parameters	Δρ_max_ = 0.76 e Å^−^^3^
0 restraints	Δρ_min_ = −0.76 e Å^−^^3^

### Special details

**Table:**

Geometry. All e.s.d.'s (except the e.s.d. in the dihedral angle between two l.s. planes) are estimated using the full covariance matrix. The cell e.s.d.'s are taken into account individually in the estimation of e.s.d.'s in distances, angles and torsion angles; correlations between e.s.d.'s in cell parameters are only used when they are defined by crystal symmetry. An approximate (isotropic) treatment of cell e.s.d.'s is used for estimating e.s.d.'s involving l.s. planes.
Refinement. Refinement of *F*^2^ against ALL reflections. The weighted *R*-factor *wR* and goodness of fit *S* are based on *F*^2^, conventional *R*-factors *R* are based on *F*, with *F* set to zero for negative *F*^2^. The threshold expression of *F*^2^ > σ(*F*^2^) is used only for calculating *R*-factors(gt) *etc*. and is not relevant to the choice of reflections for refinement. *R*-factors based on *F*^2^ are statistically about twice as large as those based on *F*, and *R*- factors based on ALL data will be even larger.

### Fractional atomic coordinates and isotropic or equivalent isotropic displacement parameters (Å^2^)

**Table:**

	*x*	*y*	*z*	*U*_iso_*/*U*_eq_	
Cu1	0.45974 (6)	1.11126 (4)	0.67569 (3)	0.02613 (17)	
Na1	0.9245 (2)	1.21836 (13)	0.62390 (12)	0.0355 (4)	
N1	0.1276 (5)	0.5042 (3)	1.1324 (2)	0.0300 (7)	
C1	0.2949 (5)	0.8902 (3)	0.8445 (3)	0.0233 (7)	
C2	0.2868 (5)	0.7638 (3)	0.8775 (3)	0.0224 (7)	
C3	0.3519 (5)	0.7198 (3)	0.7989 (3)	0.0209 (7)	
C4	0.3514 (5)	0.6022 (3)	0.8333 (3)	0.0219 (7)	
C5	0.2859 (5)	0.5315 (3)	0.9441 (3)	0.0238 (7)	
H5	0.2926	0.4528	0.9687	0.029*	
C6	0.2110 (5)	0.5782 (3)	1.0174 (3)	0.0246 (7)	
C7	0.2114 (5)	0.6939 (3)	0.9862 (3)	0.0240 (7)	
H7	0.1620	0.7239	1.0374	0.029*	
C8	0.4157 (5)	0.7967 (3)	0.6777 (3)	0.0217 (7)	
C9	0.4168 (5)	0.5492 (3)	0.7518 (3)	0.0255 (8)	
O1	0.1623 (5)	0.4011 (2)	1.1654 (2)	0.0438 (7)	
O2	0.0221 (4)	0.5489 (3)	1.1883 (2)	0.0424 (7)	
O3	0.5154 (4)	0.4541 (2)	0.7880 (2)	0.0350 (6)	
O4	0.3646 (4)	0.6026 (2)	0.6569 (2)	0.0322 (6)	
O5	0.5992 (3)	0.7895 (2)	0.6373 (2)	0.0279 (5)	
O6	0.2773 (3)	0.8631 (2)	0.62482 (19)	0.0253 (5)	
O7	0.1643 (4)	0.9349 (2)	0.8958 (2)	0.0378 (7)	
O8	0.4354 (3)	0.94091 (19)	0.76839 (19)	0.0239 (5)	
O1W	1.0173 (5)	1.3157 (3)	0.4326 (3)	0.0583 (9)	
H1W	1.1316	1.3136	0.3943	0.087*	
H2W	0.9188	1.3422	0.3956	0.087*	
O2W	0.3871 (5)	0.2405 (2)	0.8712 (2)	0.0480 (8)	
H3W	0.3277	0.2063	0.9367	0.072*	
H4W	0.4314	0.2983	0.8694	0.072*	
O3W	0.2499 (4)	0.4492 (2)	0.5859 (2)	0.0413 (7)	
H5W	0.2851	0.4832	0.6193	0.062*	
H6W	0.2712	0.4938	0.5177	0.062*	
O4W	0.8268 (4)	1.3977 (3)	0.6357 (2)	0.0457 (7)	
H7W	0.7512	1.3945	0.6941	0.069*	
H8W	0.9337	1.4228	0.6390	0.069*	
O5W	0.8772 (4)	0.8670 (2)	0.7141 (2)	0.0342 (6)	
H9W	1.0052	0.8651	0.6965	0.051*	
H10W	0.8229	0.8171	0.7068	0.051*	
O6W	0.7667 (4)	0.8945 (3)	0.9121 (2)	0.0416 (7)	
H11W	0.8793	0.9089	0.9212	0.062*	
H12W	0.7799	0.8737	0.8607	0.062*	
O7W	0.1910 (4)	1.1559 (2)	0.7608 (2)	0.0322 (6)	
H13W	0.1805	1.0929	0.8175	0.048*	
H14W	0.2254	1.1929	0.7914	0.048*	
O8W	0.6440 (4)	1.1187 (2)	0.7809 (2)	0.0304 (6)	
H15W	0.6707	1.0528	0.8295	0.046*	
H16W	0.5749	1.1599	0.8095	0.046*	
O9W	0.2728 (4)	1.0955 (2)	0.5785 (2)	0.0277 (5)	
H18W	0.2966	1.1361	0.5094	0.041*	
H17W	0.2814	1.0266	0.5904	0.041*	
O10W	0.7342 (4)	1.0795 (2)	0.5925 (2)	0.0275 (5)	
H19W	0.7623	1.0126	0.6388	0.041*	
H20W	0.7367	1.0866	0.5275	0.041*	
O11W	0.5012 (4)	1.2786 (2)	0.5697 (2)	0.0329 (6)	
H21W	0.5259	1.3005	0.5003	0.049*	
H22W	0.4304	1.3288	0.5857	0.049*	

### Atomic displacement parameters (Å^2^)

**Table:**

	*U*^11^	*U*^22^	*U*^33^	*U*^12^	*U*^13^	*U*^23^
Cu1	0.0215 (3)	0.0263 (3)	0.0308 (3)	−0.00127 (17)	−0.00157 (18)	−0.0134 (2)
Na1	0.0309 (8)	0.0395 (9)	0.0336 (8)	−0.0038 (7)	−0.0005 (6)	−0.0146 (7)
N1	0.0234 (16)	0.0362 (18)	0.0270 (16)	−0.0065 (13)	−0.0033 (13)	−0.0094 (14)
C1	0.0191 (17)	0.0243 (17)	0.0272 (18)	0.0004 (14)	−0.0049 (14)	−0.0115 (15)
C2	0.0163 (16)	0.0238 (17)	0.0279 (18)	−0.0009 (13)	−0.0026 (13)	−0.0121 (14)
C3	0.0101 (15)	0.0242 (17)	0.0283 (18)	0.0014 (12)	−0.0050 (13)	−0.0112 (14)
C4	0.0138 (16)	0.0239 (17)	0.0284 (18)	0.0013 (13)	−0.0054 (13)	−0.0114 (14)
C5	0.0200 (17)	0.0225 (17)	0.0286 (18)	0.0001 (13)	−0.0059 (14)	−0.0103 (14)
C6	0.0156 (16)	0.0294 (19)	0.0251 (18)	−0.0001 (14)	−0.0051 (13)	−0.0081 (15)
C7	0.0187 (17)	0.0280 (18)	0.0277 (18)	0.0018 (14)	−0.0023 (14)	−0.0156 (15)
C8	0.0182 (17)	0.0216 (16)	0.0286 (18)	−0.0023 (13)	−0.0015 (14)	−0.0141 (14)
C9	0.0183 (17)	0.0278 (18)	0.032 (2)	−0.0085 (14)	0.0041 (14)	−0.0158 (16)
O1	0.0474 (18)	0.0309 (16)	0.0384 (16)	−0.0047 (13)	−0.0023 (13)	−0.0029 (12)
O2	0.0423 (17)	0.0481 (17)	0.0325 (15)	−0.0075 (14)	0.0089 (13)	−0.0178 (14)
O3	0.0375 (15)	0.0285 (14)	0.0415 (15)	0.0052 (12)	−0.0052 (12)	−0.0193 (12)
O4	0.0358 (15)	0.0358 (14)	0.0275 (14)	−0.0040 (12)	−0.0032 (11)	−0.0161 (12)
O5	0.0168 (12)	0.0343 (14)	0.0294 (13)	−0.0004 (10)	0.0008 (10)	−0.0127 (11)
O6	0.0199 (12)	0.0275 (13)	0.0253 (12)	0.0016 (10)	−0.0047 (10)	−0.0090 (10)
O7	0.0351 (15)	0.0314 (14)	0.0463 (16)	−0.0050 (12)	0.0122 (12)	−0.0225 (13)
O8	0.0199 (12)	0.0218 (12)	0.0275 (13)	−0.0024 (9)	0.0004 (10)	−0.0095 (10)
O1W	0.0451 (18)	0.070 (2)	0.0423 (18)	0.0258 (16)	−0.0035 (14)	−0.0179 (16)
O2W	0.066 (2)	0.0372 (16)	0.0431 (17)	−0.0126 (15)	0.0103 (15)	−0.0239 (14)
O3W	0.0445 (17)	0.0404 (16)	0.0471 (17)	0.0023 (13)	−0.0078 (13)	−0.0270 (14)
O4W	0.0373 (16)	0.060 (2)	0.0482 (17)	−0.0100 (14)	−0.0011 (13)	−0.0305 (16)
O5W	0.0208 (13)	0.0348 (14)	0.0501 (17)	0.0011 (11)	−0.0051 (11)	−0.0219 (13)
O6W	0.0340 (15)	0.0517 (18)	0.0388 (16)	−0.0006 (13)	−0.0044 (12)	−0.0202 (14)
O7W	0.0282 (14)	0.0322 (14)	0.0372 (14)	−0.0002 (11)	0.0014 (11)	−0.0187 (12)
O8W	0.0329 (14)	0.0314 (14)	0.0302 (14)	−0.0012 (11)	−0.0060 (11)	−0.0159 (11)
O9W	0.0288 (13)	0.0267 (13)	0.0274 (13)	−0.0017 (10)	−0.0054 (10)	−0.0111 (11)
O10W	0.0232 (12)	0.0300 (13)	0.0282 (13)	0.0018 (10)	−0.0005 (10)	−0.0138 (11)
O11W	0.0394 (15)	0.0237 (13)	0.0310 (14)	−0.0019 (11)	0.0016 (11)	−0.0104 (11)

### Geometric parameters (Å, °)

**Table:**

Cu1—O8	2.028 (2)	C8—O5	1.249 (4)
Cu1—O11W	2.040 (3)	C8—O6	1.260 (4)
Cu1—O10W	2.052 (2)	C9—O4	1.247 (4)
Cu1—O9W	2.061 (2)	C9—O3	1.256 (4)
Cu1—O8W	2.086 (2)	O1W—H1W	0.8400
Cu1—O7W	2.098 (3)	O1W—H2W	0.8399
Na1—O1W	2.318 (4)	O2W—H3W	0.8400
Na1—O4W	2.422 (3)	O2W—H4W	0.8398
Na1—O8W	2.529 (3)	O3W—H5W	0.8401
Na1—O10W	2.574 (3)	O3W—H6W	0.8398
Na1—O7W^i^	2.593 (3)	O4W—H7W	0.8401
Na1—O9W^i^	2.770 (3)	O4W—H8W	0.8401
N1—O2	1.222 (4)	O5W—H9W	0.8401
N1—O1	1.224 (4)	O5W—H10W	0.8400
N1—C6	1.464 (5)	O6W—H11W	0.8399
C1—O8	1.254 (4)	O6W—H12W	0.8400
C1—O7	1.256 (4)	O7W—Na1^ii^	2.593 (3)
C1—C2	1.519 (5)	O7W—H13W	0.8400
C2—C7	1.378 (5)	O7W—H14W	0.8399
C2—C3	1.400 (5)	O8W—H15W	0.8399
C3—C4	1.400 (5)	O8W—H16W	0.8399
C3—C8	1.505 (5)	O9W—Na1^ii^	2.769 (3)
C4—C5	1.386 (5)	O9W—H18W	0.8398
C4—C9	1.521 (5)	O9W—H17W	0.8400
C5—C6	1.372 (5)	O10W—H19W	0.8398
C5—H5	0.9300	O10W—H20W	0.8395
C6—C7	1.382 (5)	O11W—H21W	0.8400
C7—H7	0.9300	O11W—H22W	0.8401
			
O8—Cu1—O11W	174.07 (9)	C2—C3—C8	121.4 (3)
O8—Cu1—O10W	89.57 (10)	C5—C4—C3	119.6 (3)
O11W—Cu1—O10W	85.25 (11)	C5—C4—C9	118.6 (3)
O8—Cu1—O9W	85.27 (10)	C3—C4—C9	121.8 (3)
O11W—Cu1—O9W	92.50 (11)	C6—C5—C4	119.6 (3)
O10W—Cu1—O9W	97.07 (10)	C6—C5—H5	120.2
O8—Cu1—O8W	91.76 (10)	C4—C5—H5	120.2
O11W—Cu1—O8W	90.54 (11)	C5—C6—C7	122.0 (3)
O10W—Cu1—O8W	83.80 (10)	C5—C6—N1	119.5 (3)
O9W—Cu1—O8W	176.89 (10)	C7—C6—N1	118.5 (3)
O8—Cu1—O7W	94.30 (10)	C2—C7—C6	118.5 (3)
O11W—Cu1—O7W	91.04 (11)	C2—C7—H7	120.8
O10W—Cu1—O7W	174.87 (10)	C6—C7—H7	120.8
O9W—Cu1—O7W	86.61 (10)	O5—C8—O6	125.2 (3)
O8W—Cu1—O7W	92.71 (10)	O5—C8—C3	118.1 (3)
O8—Cu1—Na1	118.29 (8)	O6—C8—C3	116.7 (3)
O11W—Cu1—Na1	59.92 (9)	O4—C9—O3	126.4 (3)
O10W—Cu1—Na1	49.22 (8)	O4—C9—C4	117.4 (3)
O9W—Cu1—Na1	134.47 (8)	O3—C9—C4	116.2 (3)
O8W—Cu1—Na1	48.05 (8)	C1—O8—Cu1	128.3 (2)
O7W—Cu1—Na1	125.72 (8)	Na1—O1W—H1W	128.5
O1W—Na1—O4W	90.19 (12)	Na1—O1W—H2W	115.0
O1W—Na1—O8W	146.32 (13)	H1W—O1W—H2W	114.4
O4W—Na1—O8W	91.74 (11)	H3W—O2W—H4W	104.9
O1W—Na1—O10W	89.56 (13)	H5W—O3W—H6W	105.6
O4W—Na1—O10W	134.16 (11)	Na1—O4W—H7W	116.6
O8W—Na1—O10W	65.55 (9)	Na1—O4W—H8W	107.4
O1W—Na1—O7W^i^	121.61 (12)	H7W—O4W—H8W	101.9
O4W—Na1—O7W^i^	93.88 (11)	H9W—O5W—H10W	112.6
O8W—Na1—O7W^i^	91.80 (9)	H11W—O6W—H12W	112.2
O10W—Na1—O7W^i^	124.37 (10)	Cu1—O7W—Na1^ii^	104.30 (11)
O1W—Na1—O9W^i^	76.07 (10)	Cu1—O7W—H13W	97.3
O4W—Na1—O9W^i^	139.84 (11)	Na1^ii^—O7W—H13W	118.8
O8W—Na1—O9W^i^	120.44 (10)	Cu1—O7W—H14W	107.2
O10W—Na1—O9W^i^	84.04 (8)	Na1^ii^—O7W—H14W	127.7
O7W^i^—Na1—O9W^i^	64.18 (8)	H13W—O7W—H14W	97.3
O1W—Na1—O11W	84.16 (11)	Cu1—O8W—Na1	94.13 (10)
O4W—Na1—O11W	74.72 (10)	Cu1—O8W—H15W	110.2
O8W—Na1—O11W	64.06 (9)	Na1—O8W—H15W	118.7
O10W—Na1—O11W	59.67 (8)	Cu1—O8W—H16W	105.0
O7W^i^—Na1—O11W	152.36 (9)	Na1—O8W—H16W	114.6
O9W^i^—Na1—O11W	138.74 (9)	H15W—O8W—H16W	111.7
O1W—Na1—Cu1	109.05 (11)	Cu1—O9W—Na1^ii^	99.58 (10)
O4W—Na1—Cu1	101.24 (9)	Cu1—O9W—H18W	116.8
O8W—Na1—Cu1	37.82 (6)	Na1^ii^—O9W—H18W	97.8
O10W—Na1—Cu1	37.12 (6)	Cu1—O9W—H17W	107.5
O7W^i^—Na1—Cu1	126.89 (8)	Na1^ii^—O9W—H17W	126.6
O9W^i^—Na1—Cu1	118.89 (7)	H18W—O9W—H17W	108.9
O11W—Na1—Cu1	36.68 (5)	Cu1—O10W—Na1	93.66 (10)
O2—N1—O1	124.0 (3)	Cu1—O10W—H19W	99.1
O2—N1—C6	118.0 (3)	Na1—O10W—H19W	108.8
O1—N1—C6	117.9 (3)	Cu1—O10W—H20W	118.2
O8—C1—O7	125.5 (3)	Na1—O10W—H20W	119.7
O8—C1—C2	116.4 (3)	H19W—O10W—H20W	114.0
O7—C1—C2	118.2 (3)	Cu1—O11W—H21W	121.0
C7—C2—C3	120.9 (3)	Na1—O11W—H21W	99.5
C7—C2—C1	118.4 (3)	Cu1—O11W—H22W	118.5
C3—C2—C1	120.7 (3)	Na1—O11W—H22W	118.7
C4—C3—C2	119.2 (3)	H21W—O11W—H22W	111.2
C4—C3—C8	119.4 (3)		

Symmetry codes: (i) *x*+1, *y*, *z*; (ii) *x*−1, *y*, *z*.

### Hydrogen-bond geometry (Å, °)

**Table:**

*D*—H···*A*	*D*—H	H···*A*	*D*···*A*	*D*—H···*A*
O1W—H2W···O4^iii^	0.84	2.06	2.883 (4)	166
O1W—H1W···O5^iv^	0.84	2.19	2.932 (4)	148
O2W—H4W···O3	0.84	1.94	2.706 (4)	151
O2W—H3W···O6W^v^	0.84	1.91	2.741 (4)	169
O3W—H6W···O4W^iii^	0.84	2.09	2.863 (4)	153
O3W—H5W···O4	0.84	2.01	2.825 (4)	164
O4W—H8W···O3W^vi^	0.84	2.11	2.868 (4)	149
O4W—H7W···O3^vii^	0.84	2.12	2.902 (4)	155
O5W—H10W···O1^v^	0.84	2.60	3.174 (4)	127
O5W—H10W···O5	0.84	2.05	2.778 (4)	145
O5W—H9W···O6^i^	0.84	1.89	2.711 (3)	166
O6W—H12W···O5W	0.84	2.00	2.810 (4)	161
O6W—H11W···O7^i^	0.84	1.91	2.716 (4)	160
O7W—H14W···O2W^vii^	0.84	1.98	2.788 (4)	160
O7W—H13W···O7	0.84	1.87	2.657 (4)	156
O8W—H16W···O2W^vii^	0.84	1.85	2.679 (4)	171
O8W—H15W···O6W	0.84	1.95	2.774 (4)	167
O9W—H18W···O5^iii^	0.84	1.82	2.647 (4)	168
O9W—H17W···O6	0.84	1.99	2.823 (3)	175
O10W—H19W···O5W	0.84	1.85	2.674 (4)	166
O10W—H20W···O6^iii^	0.84	1.88	2.704 (3)	167
O11W—H22W···O3W^vii^	0.84	1.85	2.670 (4)	165
O11W—H21W···O4^iii^	0.84	1.98	2.776 (4)	158

Symmetry codes: (iii) −*x*+1, −*y*+2, −*z*+1; (iv) −*x*+2, −*y*+2, −*z*+1; (v) −*x*+1, −*y*+1, −*z*+2; (vi) *x*+1, *y*+1, *z*; (vii) *x*, *y*+1, *z*; (i) *x*+1, *y*, *z*.
